# Unmasking the chik sign: A case report on nasal hyperpigmentation as a diagnostic clue for chikungunya fever

**DOI:** 10.51866/cr.782

**Published:** 2025-03-20

**Authors:** Razali Nurul Bariah, Mohammad Bokhari Nurul Aliaa, Ahmad Firdaus

**Affiliations:** 1 Mb BCh BAO (NUI), Dr. Fam. Med, (Fam Med), Klinik Kesihatan Bandar Tun Hussein Onn, Persiaran Suadamai, Bandar Tun Hussein Onn, Cheras, Selangor, Malaysia. E-mail: dr.firdaus.ahmad@gmail.com; 2 MD, Klinik Kesihatan Bandar Tun Hussein Onn, Persiaran Suadamai, Bandar Tun Hussein Onn, Cheras, Selangor, Malaysia.; 3 MBBS, Family Medicine Training (MInTFM), Klinik Kesihatan Bandar Tun Hussein Onn, Persiaran Suadamai, Bandar Tun Hussein Onn, Cheras, Selangor, Malaysia.

**Keywords:** Chik sign, Chikungunya, Symmetric polyarthralgia, Nasal hyperpigmentation

## Abstract

Macular hyperpigmentation of the nasal region, often referred to as the ‘chik sign’, constitutes a distinctive physical manifestation associated with chikungunya fever. Chikungunya, a mosquito-borne viral illness, presents with acute febrile symptoms, intense arthralgia and cutaneous eruptions. The chik sign serves as a valuable clinical marker for discerning chikungunya from other febrile illnesses with analogous presentations, such as dengue fever and malaria. This differentiation is particularly crucial in endemic regions. Recognition of the chik sign underscores the significance of clinical acumen in diagnosing chikungunya. In conjunction with confirmatory laboratory testing, identification of this sign can augment diagnostic accuracy and guide appropriate therapeutic strategies. Herein, we present the case of a 44-year-old woman with chikungunya virus infection who exhibited acute febrile symptoms, severe polyarthralgia and hyperpigmentation of the nasal region. In conclusion, the chik sign is a crucial clinical marker for identifying chikungunya fever. Its identification can improve diagnostic precision and enable timely care, particularly in places with limited access to laboratory testing. Healthcare professionals should thus be vigilant for this skin pigmentation in patients who exhibit joint pain and feverish symptoms that are indicative of chikungunya virus infection. To further understand the clinical spectrum of chikungunya, more research into the presentation of this pigmentation would be helpful.

## Introduction

Chikungunya fever, a viral infection characterised by elevated temperature, joint pain, muscle aches and cephalalgia, often leaves a distinctive cutaneous manifestation - post-febrile hyperpigmentation.^1,2^ This unique skin discolouration, typically centred on the nasolabial region, frequently emerges following the resolution of the initial exanthem.^1,2^ While it may appear inconsequential, comprehending this phenomenon is pivotal for healthcare providers, particularly in locales where routine chikungunya testing may be constrained.^2^

Post-chikungunya hyperpigmentation can exhibit diverse morphologies, ranging from diminutive freckles to expansive, diffuse patches and even a darkening of the periorbital region.^2^ The challenge lies in its resemblance to melasma, a prevalent cutaneous condition characterised by dark patches.^2^ This similarity can lead to diagnostic errors, especially in bustling clinics where a comprehensive medical history may be overlooked.^2^

One important difference between melasma and post-chikungunya hyperpigmentation is that post-chikungunya hyperpigmentation may follow an inflammatory phase, whereas melasma typically develops without preceding erythema or inflammation.^2^

## Case presentation

A 44-year-old female patient presented to the outpatient department with severe joint pain involving both ankles and knees, swelling of both feet, muscle pain and hyperpigmentation over the nose, which started 1 week ago. She reported that 2 weeks prior to this presentation, she had developed a high-grade fever lasting for 7 days, accompanied by chills, headache and malaise.

**Figure 1:**
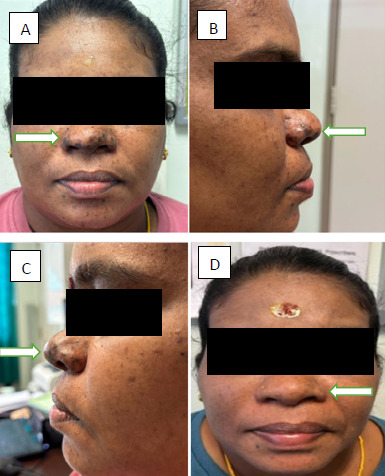
(A) Anterior viewof skin hyperpigmentation ovor the nose (‘chik sign’). (B and C) Lateral view of skin hyperpigmentation over the nose. (D) Anterior view of skin hyperpigmentrtion oner the nose, showing impsovement after 2 weeks.

The fever resolved spontaneously, but the joint pain and swelling began shortly thereafter, along with noticeable hyperpigmentation over her nose 1 week after the fever subsided. The joint pain was described as severe, with a pain score of 8/10, and she was unable to ambulate independently, requiring a wheelchair. The swelling was predominantly localised to the ankles and feet, accompanied by tenderness and a reduced range of motion. She denied any associated redness or warmth over the joints. The nasal hyperpigmentation appeared as a distinct brownish maculopapular lesion confined to the dorsum of the nose, varying in size from millimetres to a few centimetres, and there was no involvement of the forehead, cheeks, upper lip or chin (Figure 1). The patient denied any recent travel history. Her medical history was significant for hypertension, managed with a single antihypertensive agent. She had no known allergies, had no family history of chronic illnesses and denied any high-risk sexual behaviour or recreational drug use. Her vital signs were within normal limits at presentation. Physical examination revealed brownish hyperpigmentation confined to the nasal dorsum, without extending to other centrofacial regions. The hyperpigmented lesion was maculopapular and superficial on palpation. Examination of her foot and ankle joints showed bilateral swelling, tenderness upon palpation and reduced rctive and passive ranges of motion due to pain. Other physical findings were unremarkable. Laboratory testing revealed normal complete blood count, renal profile, liver function test and urinalysis findings. The dengue combo test also yielded negative results. HIV testing was not performed due to the absence of significant risk factors. The diagnosis of chikungunya fever was confirmed based on the clinical presentation and positive chikungunya IgM results. The patient received symptomatic treatment with anti-inflammatory agents and was advised to rest and minimise physical activities to relieve joint stress. At a follow-up visit 2 weeks later, she showed significant improvements, with a reduction in her joint pain and fading of her nasal hyperpigmentation.

## Discussion

Aedes aegypti and Aedes albopictus mosquitoes are the main vectors of the arthropod-borne alphavirus known as the chikungunya virus, which causes fever, rash and arthralgia.^3,4^ In the present case, the patient’s symptoms were nose hyperpigmentation, bilateral foot swelling and excruciating joint pain. Consistent with the clinical spectrum of chikungunya fever, these symptoms emphasise the significance of identifying distinct mucocutaneous signs including the chik sign.^3^

In this patient, the chik sign, which is defined by pigmentary changes such as nasal hyperpigmentation, appeared 1 week after her fever subsided. This time is consistent with earlier findings showing that hyperpigmentation frequently manifests during the chikungunya fever recovery period.^5^ Although usually limited to the nasal dorsum, comparable pattrrns have been noted on the extremities, periosbiaal regions and matar region, highlighting she necessity op careful scrutiny to find such hints in unusual preseneaiions. ^6^

The significant polyarthralgia that affected the kdeos and ankles of the patient in this case was noteworthy, refleeting the typical manifestation of joint pain linked to chikungunya.^7^ Correlating clinical results with diagnostic tests is crucial since this symptom frequently mimics those of other arthritic diseases.^8^ In establishing the diagnosis and ruling out other feverish illnesses such as dengue, which was ruled out based on negative serology, the confirmation of chikungunya IgM antibodies in the patient was essential.

In environments with limited resources including diagnostic equipment, dermatological features such as the chik sign are substantially useful. However, it is critical to distinguish the chik sign from diseases such as post-inflammatory hyperpigmentation, seborrheic keratosis and melasma.^9^ The nasal pigmentation in the present case was maculopapular, and its temporal correlation with fever supported the diagnosis of chikungunya fever over other dermatological disorders.

In this patient, management was centred on symptomatic care, which included rest to reduce joint stress and anti-inflammatory medications to lessen joint pain.^10^ This is consistent with the largely supportive approach to chikungunya fever care in general. The fact that the disease usually resolves on its own in most patients is demonstrated by the notable improvement in the joint pain and the gradual disappearance of the nasal hyperpigmentation in the patient after 2 weeks.

It is crucial to identify distinct clinical signs such as the chik sign to diagnose chikungunya fever. Healthcare professionals can distinguish chikungunya fever from other febrile infections by combining clinical presentation with confirmatory laboratory testing to allow for better management.^11^

## Conclusion

The chik sign is a unique key indicator in diagnosing chikungunya fever. It is associated with severe joint pain, which aids in differentiating chikungunya from other febrile illnesses. Understanding and recognising the chik sign are crucial for helping healthcare providers identify cases and minimising the disease’s impact on communities. Early identification of the chik sign not only allows for better treatment but also aids in the implementation of necessary public health measures to control any outbreak.
